# Encephalopathy and Neuropathy due to Glue, Paint Thinner, and Gasoline Sniffing in Trinidad and Tobago-MRI Findings

**DOI:** 10.1155/2014/850109

**Published:** 2014-06-18

**Authors:** Kanterpersad Ramcharan, Amrit Ramesar, Moshanti Ramdath, Joel Teelucksingh, Maria Gosein

**Affiliations:** ^1^Neurology Unit and Department of Medicine, San Fernando Teaching Hospital, University of the West Indies, San Fernando, Trinidad and Tobago; ^2^Department of Radiology, Port of Spain General Hospital, Port of Spain, Trinidad and Tobago

## Abstract

A 29-year-old male petrol station pump attendant was admitted with ataxia and clinical evidence of a sensorimotor polyneuropathy which developed over the preceding 3 months. He had cognitive dysfunction, hearing loss, and cerebellar clinical abnormalities that came on slowly over the three years. He had a fifteen-year history of sniffing mostly glue, occasionally paint thinners, and, in the recent two years, gasoline. Magnetic resonance brain imaging showed abnormalities of the cerebral cortex, cerebral white matter, corpus callosum, hippocampus, brainstem and cerebellar atrophy, hypointensities of basal ganglia, red nuclei, and substantia nigra as previously described in toluene sniffing. Abstinence for six months led to partial clinical improvement. Clinicians need to be aware of this preventable entity which has peculiar radiological findings which are being increasingly accepted as typical.

## 1. Introduction

Toluene toxicity from glue, paint thinners, and petrol (gasoline) sniffing is being recognized globally and reports documenting the neurotoxic effects of this practice have focused on the multisystem nervous system presentations, the pathogenesis of which is still unclear [1, 2]. Peculiar abnormalities on magnetic resonance imaging, albeit not being common except in severe cases, have been reported repeatedly [3–8]. Inhalant substance abuse has not been a common practice in the West Indies. We report a young man who was introduced to this form of substance abuse at age of 15 by a friend from the United States and presented to hospital 15 years later with ataxia, cognitive abnormalities, and peripheral neuropathy.

## 2. Case Report

A 29-year-old-Afro-Caribbean male presented with a three-month history of difficulty in walking with numbness and weakness of the hands and feet which had worsened over the previous 3 months. He had to be supported to walk on admission. The accompanying brother noted that he had difficulty with speech, reading, and writing which had been noted a few years before.

He had no chronic illnesses, had no previous surgery, was single, and lived with his parents. He was not a vegetarian and worked as a petrol station attendant. He did not use alcohol, cannabis, tobacco, or cocaine. There was no family history of consanguinity or neurological illness. Further questioning as to the possibility of heavy metal poisoning led to a history of glue, paint thinners, and more recently petrol sniffing at his workplace.

For most of the 15 years, glue sniffing was the predominant form of inhalant abuse. He had been introduced to the pleasure of glue sniffing at age of 15 by a friend who was visiting from the USA and had abused glue and to a lesser extent paint thinners and gasoline since working as a gas station attendant. He had no bowel or bladder dysfunction but admitted to diminished vision and hearing for about a year.

Physical examination showed an underweight slim built man of 5 feet 9 inches, weighing 155 lbs, who walked with a high steppage gait. Vital signs were normal. The chest, cardiovascular, abdomen, locomotor, and endocrine systems were normal. He was conscious with dysarthria and slowed speech and thought with mild hearing deficit. He did not know the date but knew the place and person and scored 26/30 on Mini Mental status scale. There was horizontal nystagmus in both eyes at the extreme of right and left conjugate gaze. Also abnormal were finger to nose and heel on shin tests bilaterally and abnormal rapid alternate movement test in both hands and feet. The Romberg test was positive. Reflexes were absent at knees and ankles with downgoing plantar reflexes bilaterally and power was 4/5 in all motor groups. There was a glove and stocking sensory loss to light touch, pinprick, and vibration sense without apraxia or agnosia. Asterognosis was normal in the hands.

Investigations revealed that lead levels, urine for porphyrin, serum B 12, red cell folate, T4, TSH, T3, urine microscopy, HIV 1 and 2 by Elisa, HTLVI, FBC, creatinine, liver function, c ANCA, p ANCA, ANA, C reactive protein, serum protein electrophoresis, ESR, and serum magnesium were all normal. CPK was mildly elevated at 600 IU/L. Chest X-ray, CT scan chest abdomen and pelvis, and EEG were normal. Electromyogram/nerve conduction study showed slowed delayed distal motor latencies and decreased conduction velocities in the median, ulnar left deep peroneal, and left posterior tibial nerves. A sensory nerve conduction study was not done. Audiometry showed sensory neural partial deafness in the right ear. Consent for a spinal tap was not obtained.

**Figure 1:**
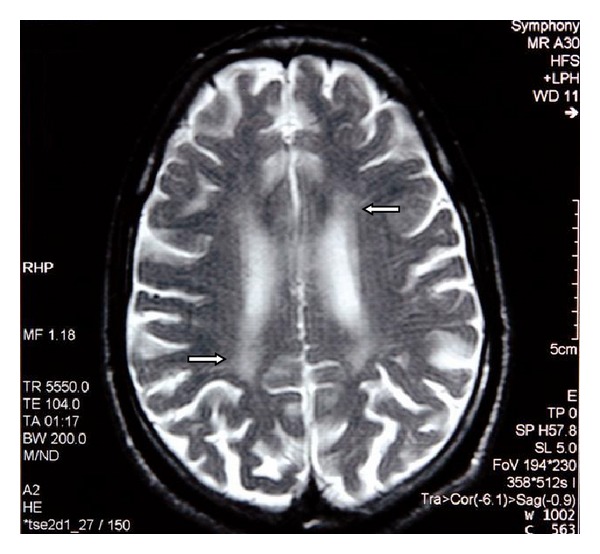
Axial T2 weighted MR image of the brain shows generalized cerebral atrophic changes with associated prominence of cortical sulci. There are high signal intensity changes in the periventricular white matter (arrows) along with some loss of grey matter-white matter differentiation.

**Figure 2:**
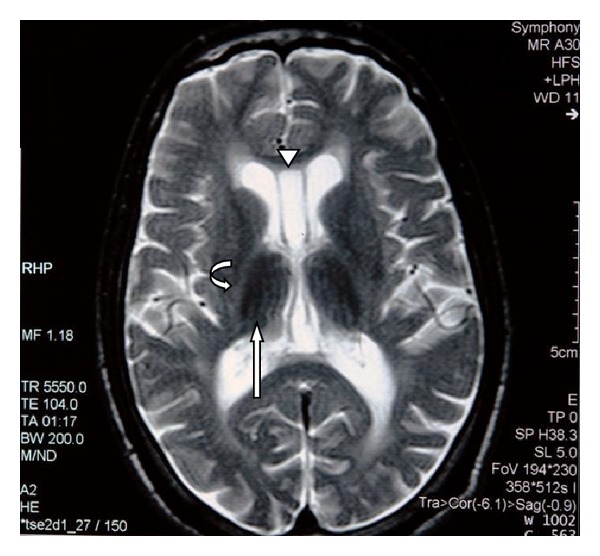
Axial T2 weighted MR image of the brain at the level of the basal ganglia. There is symmetric hypointensity of the thalami (straight arrow) as well as hyperintensity of the posterior limbs of internal capsule (curved arrow). Cerebral atrophic changes also noted with rounded horns of lateral ventricles. Normal variant cavum septum pellucidum and vergae noted (arrow head).

**Figure 3:**
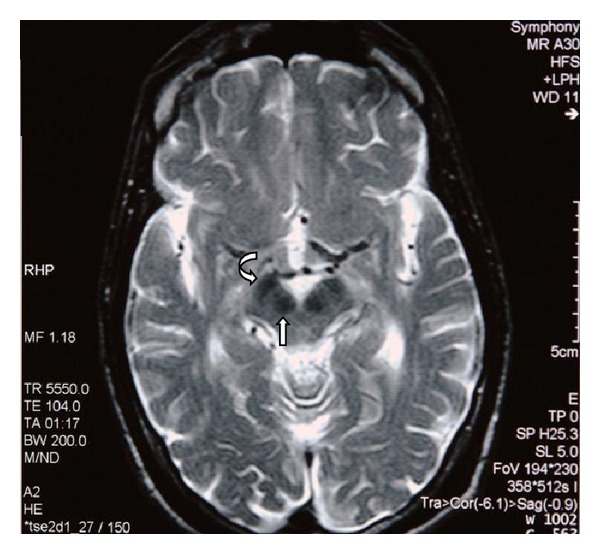
Axial T2 weighted MR image of the brain at the level of the midbrain reveals hypointensity of the red nuclei (straight arrow) and substantia nigra (curved arrow).

**Figure 4:**
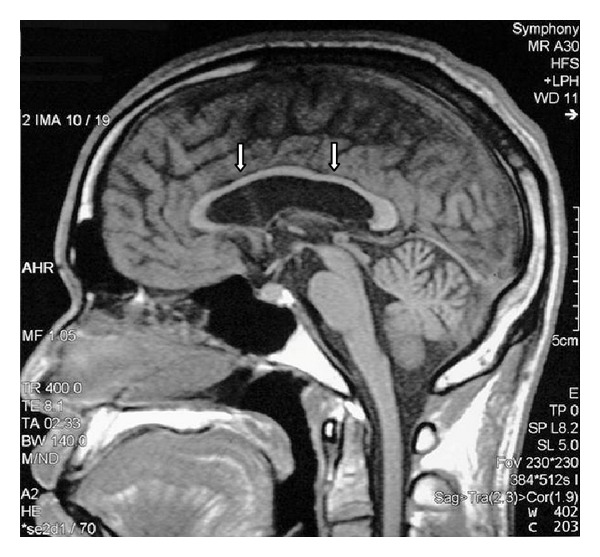
Sagittal T1 weighted MR image of the brain shows thinning of the corpus callosum (arrows). Cerebellar atrophy also evident with enlargement of cerebellar sulci.

**Figure 5:**
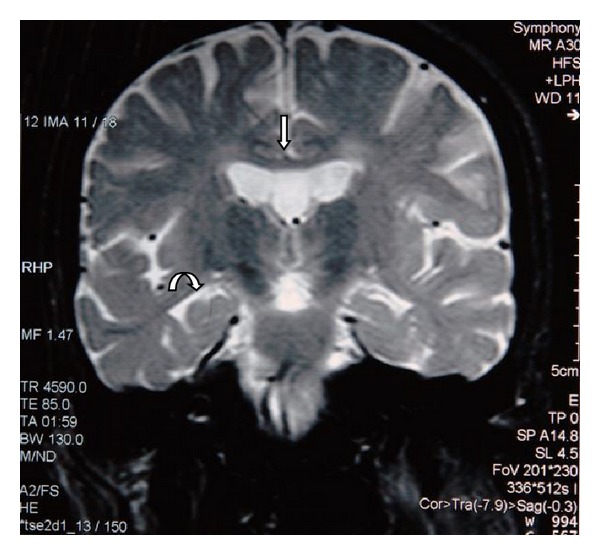
Coronal T2 weighted MR image of the brain shows bilateral hippocampal atrophy (curved arrow). Thinning of the corpus callosum also noted on this image with no evidence of signal intensity change (straight arrow).

MRI of the brain revealed generalized atrophy with high T2 signal intensity changes of the periventricular white matter and some loss of grey matter-white matter differentiation (Figure 1), T2 hypointensity of the thalami and hyperintensity of the posterior limbs of internal capsule (Figure 2), and T2 hypointensity of the red nuclei and substantia nigra (Figure 3) as well as thinning of the corpus callosum and hippocampal and cerebellar atrophy (Figures 4 and 5). As sufficient diagnostic information was obtained on noncontrast study and due to increased cost factors involved, postgadolinium images were not obtained. There were no abnormalities detected on MRI of the spine which showed normal morphology and MR signal of the spinal cord.

**Figure 6:**
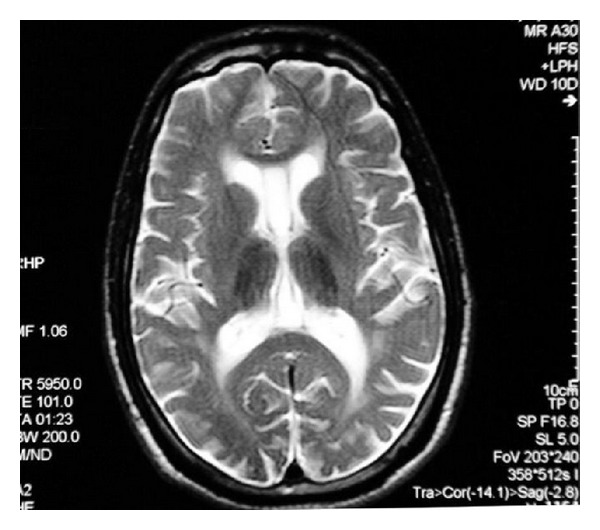
Interval axial weighted MR image of the brain following a six months of abstinence shows no significant interval change in the thalamic hypointensity or the periventricular white matter and posterior internal capsule hyperintensity, despite clinical improvement.

Over the next six months abstinence resulted in global recovery with disappearance of the high steppage gait, nystagmus, quicker thought processes, coherent speech, and ability to read and write fluently. Cerebellar signs were still present. Mini Mental status scale had improved to 30/30. However, follow up MRI after 6 months of abstinence showed no significant interval change in T2 thalamic hypointensity or white matter and internal capsule hyperintensity, despite clinical improvement (Figure 6).

Repeated nerve conduction study at 6 months following abstinence showed delayed distal motor and sensory latencies in the left median and left superficial peroneal nerves and delayed motor distal latencies in the left deep peroneal and posterior tibial nerves. There had been mild improvement from the previous study. Electromyogram showed no evidence of chronic denervation in the left thenar, hypothenar, extensor digitorum brevis, anterior tibialis, and abductor hallucis muscles, but there was mild improvement in the interference pattern. The CPK dropped to normal level at 6 months.

## 3. Discussion

Our patient admitted to abusing mostly glue and petrol and thinners to a lesser extent. The typical MRI findings are shown in Figures 1–5 and are consistent with multiple previous reports of this entity [3–8]. Our patient abused vapors which would have a mixture of hydrocarbons but predominantly toluene and hexane. With toluene found in glue sniffing, the MRI findings of white matter changes and the prominent nuclei of the basal ganglia and brainstem are well documented [8].

Whilst the deafness and optic atrophy have been seen in toluene toxicity of glue sniffing, the peripheral neuropathy could be attributed to hydrocarbons including toluene in gasoline and toluene of glue [9–14]. The combination of encephalopathy with cranial and peripheral neuropathy is also documented in glue sniffers [13]. Peripheral neuropathy has also been described in glue sniffers predominantly being motor with sural nerve biopsy abnormalities [12].

The pathogenesis of increased water content of the white matter or toluene-induced metabolic changes in myelin have been postulated to explain the white matter MRI changes and diffuse myelin pallor, preservation of neurons, minimal gliosis, and scant perivascular macrophages at post mortem [4]. In a postmortem study done from Mexico demyelination and diffuse axonopathy with axonal loss were documented in the brain [11]. Gasoline sniffing whilst being an international problem has a higher prevalence in ethnic minorities.

It is prevalent in Australia, Northern Canada, and Southwestern USA and causes cognitive and neurological deficits in chronic sniffers [9, 15]. Gasoline has many hydrocarbons inclusive of toluene [16]. Peripheral neuropathy of axonal and demyelinating type has also been noted in gasoline sniffing and may have been the cause in our patient [2]. Elevated serum creatinine kinase has been noted in sniffers of leaded and unleaded gasoline and has been suggested as being useful in detecting current petrol sniffing in locations using unleaded gasoline [17]. In Trinidad and Tobago only unleaded gasoline has been used since the year 2000.

This problem has not been recognized as a major problem so far in the West Indies. Our patient has stopped abusing substances for six months and has been referred to a substance abuse clinic. The improvement as noted in our patient has been noted in rats exposed to toluene inhalation [18]. The improvement after 6 months of abstinence has been modest and we hope to monitor the patient prospectively using clinical, imaging, and electrophysiological studies.

We hope by this report to inform clinicians of the deleterious effects of volatile substance abuse on the nervous system, maintain a high degree of suspicion in taking a recreational drug history, and highlight the MRI changes noted.
